# Successes and challenges of speech language therapy service provision in Western Kenya: Three case studies

**DOI:** 10.4102/sajcd.v68i1.838

**Published:** 2021-09-27

**Authors:** Bea Staley, Ellen Hickey, David Rochus, Duncan Musasizi, Rachael Gibson

**Affiliations:** 1 College of Indigenous Futures, Education and the Arts, Charles Darwin University, Darwin, Australia; 2 School of Communication Sciences and Disorders, Dalhousie University, Halifax, Canada; 3 Yellow House Health and Outreach Services, Kisumu, Kenya

**Keywords:** Speech language therapy, Kenya, collaboration, service delivery, sustainability

## Abstract

**Background:**

The need for communication-related services in sub-Saharan Africa to support individuals experiencing communication disability is a longstanding and well-documented situation. We posit the inequities highlighted by coronavirus disease 2019 (COVID-19) make this a relevant time for speech language therapists and the professional bodies that govern us to broadly consider our roles and practices in education, health and disability in local, national and global contexts.

**Objective:**

To illustrate what services developed with local knowledge can look like in Kenya in order to promote dialogue around alternative speech language therapy models, particularly in contexts where there are insufficient services, few trained speech language therapists and limited structures to support the emerging profession.

**Method:**

This article examines three clinical case studies from Western Kenya, using a conceptual framework for responsive global engagement.

**Results:**

Service needs in Western Kenya well exceed a direct one-on-one model of care that is common in the minority world. The service delivery models described here emphasise training, skills sharing and engaging the myriad of communication partners available to individuals with communication disabilities.

**Conclusion:**

We offer up these case studies of collaborative practice as contextual realities that may be present in any speech language therapy programming in under-resourced communities. We dispel the idea that success in this work has been linear, progressed on planned time frames or come to fruition with targeted goal attainment. The fact that our relationships have endured in these communities since 2007 is our primary success.

## Background and rationale

The World Report on Disability (WHO, 2011) estimates that more than a billion people live with disability, including 95 million children, and that disability prevalence figures are increasing due to chronic health conditions. Hartley and Wirz (2002) estimated that the proportion of people with communication disability is somewhere between 25% and 49% of this population, while Olusanya and Okolo (2006) inferred that about 40 million of the estimated 115 million children not attending school globally have communication disorders and other disabilities. There is a notable discrepancy between the prevalence figures for individuals with disability and the rehabilitation services available (WHO, 2011). The dearth of speech language therapy (SLT) services in sub-Saharan Africa in relation to the region’s population, however, is certain (Bunning et al., 2014; Wylie et al., 2016b), as Khoza-Shangase and Mophosho (2021) state we have a ‘known demand versus capacity challenge’ (p. 1). This remains despite the knowledge that communication access is a stated human right (McLeod, 2018; United Nations General Assembly, 1948).

The global COVID-19 pandemic has made explicit the interconnected nature of the planet. We have seen and felt local impacts on our own well-being and communities due to global events and decision-making. How international governments responded and reacted to the COVID-19 outbreak had a palpable effect on our daily lives. We have also seen, via the media, the inequity of healthcare service availability and accessibility in both minority and majority world contexts (e.g. Roy, 2020; Yashadhana, Pollard-Wharton, Zwi, & Biles, 2020).

It is a relevant time for speech language therapists and the professional bodies that govern us to broadly consider our roles and practices in education, health and disability in local, national and global contexts. If communication is a human right, and speech language therapists possess the skills to meet the needs of individuals with communication difficulties, there is surely an obligation to critically engage in conversations to ameliorate the service disparities in our communities and around the world (Hartley, 1998) to re-imagine and re-vision the possibilities for the profession (Khoza-Shangase & Mophosho, 2021). As McLeod (2018) notes, ‘the importance of communication rights goes beyond just enabling freedom of opinion, expression and language. Once these rights are realised, people are more readily able to realise other human rights’ (p. 4).

The development of more communication-related services in sub-Saharan Africa to meet the needs of individuals with communication disability is a longstanding, documented need (Hartley & Newton, 2009; Olusanya, Ruben, & Parving, 2006; Pillay & Kathard, 2018; Wylie, McAllister, Davidson, & Marshall, 2013). This need is currently being met in various ways, including the visible growth and presence of the profession in countries like Zambia (Bright & Selemani, 2017), Uganda (Barrett & Marshall, 2013; Marshall & Wickenden, 2018), Kenya (e.g. Staley et al., 2019), Rwanda (Mukara et al., 2017) and Ghana (Wylie et al., 2016a, 2017).

The provision of services is also being met through a proliferation of minority world – majority world partnerships (e.g. Staley et al., 2019, 2021; Wylie et al., 2016a). However, authors such as Khoza-Shangase and Mophosho (2021) and Pillay and Kathard (2018) urge us to critically consider how SLT services are provided to better meet the needs of individuals experiencing communication disabilities in African contexts. They highlight that many of the services currently offered are colonising: a reduplication of minority world cultural ideals, structural racism, language socialisation and practices delivered in transported models of ‘service exportation’ (Hartley, 1998, p. 278).

The purpose of this article is to illustrate what services developed with local knowledge can look like in Kenya. The aim of this article is to promote dialogue around SLT practices, particularly in contexts where there are scant services, few trained speech language therapists and limited structures to support the emerging profession.

## A framework for revisioning speech language therapy services

Pillay and Kathard (2018) as well as Hyter (2014) propose frameworks for the SLT profession, challenging traditional conceptualisations of the field, which are based on epistemologies and theories developed around white middle class experiences and understandings of the world. Here, the authorship team describe our ongoing clinical collaboration between minority and majority world speech language therapists. We use Hyter’s framework (2014) for responsive global engagement to share work that occurred in Western Kenya between 2007 and 2020.

Hyter’s (2014) framework guides speech language therapists to consider social structure factors that influence the availability of and access to SLT services and to be aware of and to consider diverse worldviews in our clinical practice. Hyter (2014) provides a structure for thinking about the contextual factors that impact the outcomes of our services, particularly for clients from culturally and linguistically diverse backgrounds, and to consider the factors that impact availability and sustainability of SLT services in global contexts or marginalised groups in our own countries (Hyter, 2014; Pillay & Kathard, 2018).

Hyter (2014) posits that in order to develop sustainable and culturally relevant SLT services with groups of people from culturally and linguistically diverse backgrounds, we must develop a better understanding of the macro level social structures that impact people (e.g. historical, cultural, economic, and political factors). This broader understanding, or world literacy, allows clinicians to take a broader view of clients’ and families’ contexts. The result is responsive global engagement, which is ‘an ongoing process of self-reflection and reciprocity’ (Hyter, 2014, p. 115) that involves collaborative and sustainable work with communities from different parts of the world.

Furthermore, there are four constructs associated with the process of responsive global engagement: global humility, global self-awareness, global knowledge and global reciprocity (Hyter, 2014). These constructs promote self-awareness of our own cultural assumptions and views, as well as an openness to learning about other cultures, knowledge and the contextual conditions of our clients and families. They guide us in modifying our practices to better match the client and family’s beliefs and values, to build more relevant and sustainable SLT services. That said, it is necessary to understand that responsive global engagement is a process rather than an endpoint and depends on the iterative work of theory, collective direct action, critical reflection, creation of new knowledge and practice change and so on in cyclical fashion (Hyter, 2014).

This article examines three clinical case studies, using Hyter’s conceptual framework for responsive global engagement (see Hyter, 2014; Hyter & Salas-Provance, 2019) to elucidate our thinking about the local context, practices and key elements in our ways of working. This framework supports explicit thinking about power, epistemology of practice and the best ways to meet the needs of children and families in Western Kenya’s given macrostructural factors within the context.

These case studies were generated from our collective memory of each project and a narrative lens where ‘meaning is constructed through story’ (Ely, Vinz, Downing, & Anzul, 1997, p. 63). We understand that through the iterative process of reflecting, writing and editing this narrative together as a group of collaborators with varying viewpoints and worldviews, we make deeper meaning of our work and understand better our working relationships as clinicians and authors. Thus, this article is a co-construction of local and global knowledge that came out of conversations and work among the authors. Through writing, we understand better each other’s perspectives, goals and intentions (Ely et al., 1997).

## Research questions

Although qualitative in nature, this work intended to answer the following questions:

- What do we learn about the successes and failures of our SLT work in Western Kenya by analysing case studies of our practice?
- How can we use this information to improve clinical practices?
- How do we ensure that the resources, perspectives and knowledge of all partners is being fully utilised to benefit individuals’ experiencing communication disability?

## Method

### Case study design

A qualitative case study design (Stake, 2005) was used to examine the three different types of work being engaged in by one non-governmental organisation (NGO) in Western Kenya. Case study is not ‘a methodological choice but a choice of what is to be studied’ (Stake, 2005, p. 443). Cases are necessarily bound by time, place and activity (Stake, 2005).

We explored these three SLT cases, which describe collaborations between one NGO that supports SLT development and three government-funded health and education institutions that took place between 2007 and 2020 (see Table 1). We describe the locales, projects and their trajectories to date and share our experiences as collaborators in SLT service provision over the years. The case studies of collaborative practice presented here were developed to both contribute to the literature on what SLT practices can look like in situ as well as provide the authorship team with an opportunity for reflection, discussion and improvement.

**TABLE 1:** Characteristics of case studies.

Pseudonym	Clinical models	Sector
**Case 1**		
Kilimi Children’s CommunityCentre	One-on-one therapy, community outreach and education, parent and teacher training, classroom observations.	Education/Disability
**Case 2**		
Ziwa Provincial Hospital Kijiji District Hospital	One-on-one therapy, participation in hospital rounds, patient support groups, inter-professional training, interdisciplinary co-treatment.	Medical/Hospital
**Case 3**		
None, bespoke service models: Parent Liaison Officer Communication Camp	Community outreach and education, parent training, parent support networks, case management, individualised therapy plans.	Community

This case study approach (Stake, 2005) paired with Hyter’s (2014) conceptual framework for responsive global engagement were used to explore the collaborative partnerships for developing SLT services in Western Kenya. This approach was used because (1) we could not control or manipulate the cases studied; indeed these cases are presented in retrospect and (2) we aim to describe the contextual conditions and how/why each of the projects was more or less successful in developing SLT services (Baxter & Jack, 2008; Yin, 2003).

Qualitative data from these reflections were triangulated with data from email communication, files, organisational records and conversations amongst the clinicians and researchers about the SLT projects over the past 13 years. As we created the case descriptions, reviewed documents and recollections, we came to better understand the impact of collaboration on our activities. Furthermore, this collaborative writing process was an opportunity for bi-directional, culturally responsive learning (Hyter, 2014).

### Ethical considerations

This article followed all ethical standards for research without direct contact with human or animal subjects.

## Results

### Case 1: Collaborating with government funded education services

Kilima Children’s Community Centre (all place names are pseudonyms) was a project grounded in a prior working relationship between a foreign but locally based speech language therapist (the first author on this article) and a community professional trained to work in the Kenyan disability sector. The team at the government-funded education centre wanted to expand their centre’s service capabilities based on the community’s needs. Specifically, they wanted a space that caregivers and their children could comfortably gather for therapy services and information sharing. In 2007, this team led the process to renovate an onsite government building that was being used intermittently to store grain. The team identified a grant opportunity from a Kenyan telecommunication company to fund the refurbishment and led the application process, while the speech language therapist’s role was encouragement and editorial. The grant provided USD $20 000 to repair the roof, windows and doors to transform the building into a community therapy centre for children with disabilities. A second grant was obtained by the speech language therapist from a consulate in Nairobi to build two wheelchair accessible pit latrines, as well as to buy resources, therapeutic equipment and furniture. The building was then used for a cerebral palsy clinic, occupational therapy (OT) services and, later, SLT services.

Because of the funding needed to support the new services, a local Community Based Organisation (CBO) was established to run the project. The CBO worked in partnership with an international NGO that was set up in the United States to fund monthly operating costs, including utilities for the building, salaries for the occupational and speech-language therapists, fuel for the motorbike for community outreach and funds for parent trainings. Though the funding for the Kilima project has largely come from overseas fundraising, the way services have been provided has always been informed by the needs of the community and within the boundaries of a collaborative relationship. This has not always worked, however, in terms of continuity of care.

In 2012, a change in leadership at the district level resulted in a change in local administrative staffing and the collaboration ended swiftly and with tension. Then in 2017, the partnership was restoked tentatively when local administrative staffing changed once again. During the interim, when allied health services were not available, parents who lived in the area reportedly kept requesting the services that they had seen emerging in their district between 2012 and 2017 prior to the dissolution of the collaboration.

The relationship between the speech language therapists working for the NGO and colleagues working at Kilima Children’s Community Centre was rekindled when the SLT team approached the Kilima team and requested the use of the Centre to see an adult client who was unable to make the long trip from their home to the speech language therapist’s practice. The team willingly agreed and requested that SLT services be made available for any clients on the Centre’s waiting list. Since that time, the NGO provides 1 day of SLT services at the Centre per week under a Memorandum of Understanding.

Ongoing stigma and shame around disability in Kilima present as a challenge for Kilima Children’s Centre. However, this community has a strong network of involved parents, and the physical location of Kilima Children’s Community Centre and access to public transport makes it an ideal place for clinical service provision and trainings. The staff at the Centre have been working to shift local attitudes to reduce shame associated with having a child with a disability.

In the years that this collaboration has both worked and not worked, there have been notable impacts at the client/family and community levels, including a growing awareness about the SLT profession and the scope of SLT services. Kilima Children’s Community Centre staff reported several improvements, as follows. At the client/family level, more families coming to the centre for assessment and families receiving services have reported increased confidence in their ability to support their child’s development. At the community level, improvements were made for data keeping for children with disability in the county by centre staff.

Stigma and shame around disability appear to have reduced in the community per staff report, though no measurable indicators for this are available. Recent cooperation from county officials in education has been unprecedented, enabling teachers to attend trainings around communication disability and extending SLT education support for street connected children with special education needs and communication disabilities. Further, word has travelled, and children are often referred from neighbouring counties.

The partnership is far from perfect, however. One difficulty is finding ongoing funding to support the community-based work that speech language therapists and the Kilima Children’s Centre staff used to do jointly. Further, for a variety of reasons (e.g. less time available for meeting, increased caseloads), there is less collaboration between the two organisations with individual clients, so families no longer receive an interprofessional rehabilitation approach.

### Case 2: Collaborating with hospital services

In 2007–2008, a foreign-locally-based speech language therapist (B.S.) and a local hospital-based occupational therapist initiated a transdisciplinary relationship that allowed for SLT service provision within the OT department at the provincial hospital in Ziwa. In return for SLT service provision for their outpatient referrals, the OT department provided a small room to serve as a therapy room and office. The presence of a speech language therapist presented opportunities for in-house professional development about communication disabilities and SLT practices.

The NGO’s involvement in hospitals grew over time, driven by speech language therapists (D.R., D.M. and R.G), who provided SLT trainings and clinical services in the hospital 1 day per week. Working inter-professionally with occupational therapists afforded an opportunity to build awareness of the SLT scope of practice and the services that could be offered appropriately to patients. Since late 2015, the NGO has funded an SLT position within Ziwa Hospital 2 days per week. However, increase in referrals has caused staffing pressures, as the NGO has a finite number of clinical days assigned to cover both in- and out-patient units, and this impacts the dosage and intensity of service provision available for clients.

The inter-professional partnership between the SLT and OT departments has been mutually beneficial, providing for collaborative management of children with a variety of diagnoses, creation of a support group for stroke patients with aphasia and management of swallowing and feeding difficulties. Over time, this collaboration has extended to supporting OT and clinical psychology students from the Training College on their rotation through the OT department. In turn, the hospital occupational therapists have provided similar individualised support to international SLT students on clinical placement with the NGO.

Another neighbouring hospital collaboration was successful to start, but not successful in the long term. Between 2012 and 2016, the NGO worked in Kijiji, a rural district hospital. In its prime, this inter-professional collaboration allowed speech language therapists the opportunity to liaise with hospital-based occupational therapists, physical therapists, nurses, nutritionists, doctors, clinical officers, the orthopaedic technologists and biomedical engineers. The speech language therapists developed relationships with individual nurses, who over time learned what speech language therapists do and started referring in-patients with feeding difficulties or those experiencing communication disability. The speech language therapists were able to leave feeding plans for nurses or the family to follow. They were also able to work with nutritionists around dietary needs and types of food clients would eat. The speech language therapists also referred clients for malnutrition and later the nutritionists referred clients with feeding difficulties.

Moreover, speech language therapists referred clients who presented with epilepsy, fevers or acute medical conditions to the doctors and clinical officers on site. A Parent Liaison Officer (employed by the NGO) would follow up on these referrals with parents, alleviating any anxiety about the appointment and serving as a case manager for the family. The Parent Liaison Officer also supported the parents to implement any recommendations and strategies and reminded families of upcoming appointments. This partnership ended unceremoniously when the space allocated to SLT services was reallocated to a county government official, and the clinic equipment and furniture were found piled up in the hospital corridor.

Although this work involves three government hospitals in the region (two relationships described here), the work is funded by international donations. This is due to national issues around the development of the SLT profession in Kenya and lack of government recognition while the Association of Speech and Language Therapists Kenya works towards a scheme of SLT service, or an agreement around the inclusion of SLT services into the hospital’s patient billing system. Because the work does not generate revenue or cover staffing costs, resourcing a hospital-based service remains an ongoing challenge.

That said, the benefits of hospital-based relationships are undeniable. Speech language therapists’ contributions to a rehabilitation team are contextualised, resulting in increased awareness and referrals for SLT services. Communication between the speech language therapists and the hospital administrators improved over time, allowing access to wards and clinical space, resulting in invitations to Continued Medical Education meetings attended by most medical and rehab staff. This increased professional visibility of speech language therapists and consequently increased awareness of communication and swallowing disabilities and an increase in appropriate referrals to SLT services. Ward nurses and doctors directly started referring in-patients to speech language therapists.

### Case 3: Collaborating with the community

Two elements of the clinical SLT work that have developed successfully and innovatively in the past few years are the creation of a position of Parent Liaison Officer and the implementation of a community-based service model known as Communication Camps (Chance for Childhood, 2015). These are presented as a third case study because they are co-occurring practices. Both the position of Parent Liaison Officer and the Communication Camps have positively impacted the community relationships and case studies already described in this article.

#### Parent Liaison Officer

The creation of a Parent Liaison Officer position evolved out of a relationship between the NGO’s speech language therapists and a parent of a child with disabilities who had been receiving SLT services. The parent was hired as a Parent Liaison Officer to work with parents alongside the team’s speech language therapists. The Parent Liaison Officer’s many roles were designed for a person who could speak to first-hand experience with SLT services. The Parent Liaison Officer works with parents who are referred for SLT services and she attends SLT clinics to support parents and answer their questions. The Parent Liaison Officer runs parent support groups for parents of children experiencing communication disability and uses her social capital, community connections and networks to reduce the stigma around communication disability. She also case manages for families engaged with hospital-based services and helps to identify children that may have been overlooked for referral.

Perhaps most importantly, the Parent Liaison Officer engages in the crucial work of expectation management. Caregivers are often looking for a cure and expect that the speech language therapist will be able to ‘fix’ their child, make them talk or promote the immediate recovery of a child’s speech. When this does not happen, there can be confusion about the purpose and process of SLT practices. This Parent Liaison Officer role has been transformative for the organisation by increasing parent buy-in and the number of return appointments and new referrals.

#### Communication camps

The NGO has also begun training parents of children with disabilities through a service delivery model called Communication Camps (Chance for Childhood, 2015). Communication Camps are for parents and children with severe communication disability and/or eating and swallowing difficulties. Families are invited to participate when they have limited access to health and therapy services, have not had a similar training before and have a willingness to learn more about their child’s disability and associated difficulties. Often the children are identified through Community Extension Health Volunteers, Kilima Children’s Community Centre, the out-patient department of Kijiji hospital and word of mouth. Referrals often exceed the capacity of the team.

The Communication Camp training curriculum was developed by and is continually refined by the speech language therapists and the Parent Liaison Officer based on experiences and stakeholder feedback. Communication Camps are funded through international grants and attended by parents, children, two speech language therapists and the Parent Liaison Officer. Communication camps cost USD $150 per family. This cost includes the 2-day camp costs, initial identification of the child and family and 20 follow-up visits of the child and parent as part of a support group.

To date, the teams have run 15 Communication Camps for 107 caregivers of children with disabilities. After the Communication Camp, caregivers are provided follow-up support by the Parent Liaison Officer. Follow-ups are home visits, support group visits or phone calls. The speech language therapist work with the family intermittently, providing strategies that supported the child’s progress.

The Communication Camps have been one of the most successful and innovative programmes trialled by the NGO to meet the needs of children experiencing communication disability in the community. Feedback from the Communication Camps and parent support group is that parents are better able to view their child as more than their disability, as well as understand their child’s disability and how this impacts their child’s participation in the community. The success of the NGO funded camps has been due to the multifaceted approach from the Kilima Children’s Community Centre team, the Parent Liaison Officer who works with the parent support group and the speech language therapists provided by the partnering NGO.

## Discussion

Service needs in Western Kenya well exceed a direct one-on-one model of care that is common in the minority world (Hyter, 2014; Pillay & Kathard, 2018). The community contexts described here necessitate service delivery models that emphasise training, skills sharing and engaging the myriad of communication partners available to individuals experiencing communication disabilities. Optimally, all SLT clinical service models are developed in response to community context and need (Barrett, 2016; Hyter, 2014) ‘or ‘contextual realities’ (Khoza-Shangase & Mophosho, 2021, p. 4). Locally developed service delivery can account for the structural factors in place, including historical, political, economic and cultural structures that present barriers or supports for development of SLT services (Hyter, 2014; Khoza-Shangase & Mophosho, 2021).

These case studies describe some of the successes and failures in developing SLT services in Western Kenya and have been interpreted using Hyter’s (2014) framework of responsive global engagement. We structure the discussion by answering our research questions:

1. What do we learn about the successes and failures of our work in Western Kenya by analysing case studies of our practice?

At the Kilima Children’s Community Center, clinical services evolved over time, in response to varying factors. A need was identified within the local context and services developed based on the preferences of the client group (families of children with disabilities), taking into account the social factors that made this a desirable context for services (Hyter, 2014). Despite strong relationships in the community and an understanding of the historical and socio-cultural context, however, the SLT services at the Kilima Children’s Community Centre stumbled.

Changing political landscapes and administrative positions will always be a challenge when working outside of governmental health and education structures. Even when collaborative interprofessional practice is working well, economic and political factors can impact the sustainability of clinical work. Local social structures supported the growth, the demise and then the redevelopment of this collaborative project. Kilima is an optimistic case study for our team, as it demonstrates that over time some projects may cease then later resume, possibly in a new format and with new stakeholders.

As an NGO and partnering organisation, staff report that they could not have foreseen the varying dynamics that ended the partnership between the organisations in 2012. But, political factors, such as changes in governmental positions at district and regional levels, can completely shift the context, so that active and viable projects become untenable.

The demise of the collaboration at Kijiji hospital was also related to macrostructural or institutional administrative changes outside of the collaboration and largely unrelated to the clinical work. Hospitals are highly contextualised and bureaucratic places and support within the administrative structures is essential for the development and continuation of any successful partnership.

2. How can we use this information to improve clinical practices?

Our noted successes and failures, big and small, are a part of an ongoing and somewhat cyclical story as per responsive global engagement (Hyter, 2014). A programme went well until it no longer did; changes were made, and things improved, or they did not. We explore both aspects of the work, so that the lessons learned and the processes we engaged with can inform not only ourselves, but other practicing clinicians working in similar contexts. These case studies demonstrate the need to build flexibility into the way service models emerge.

Developing SLT services in rural East African contexts depends on the skills, attitudes and motivations of the individuals available in a community at any one time. In these cases, local context was imperative and SLT options were determined by the available space, expertise, support and overall energy for SLT service provision in the community. The need in the region is extensive, but the reason this NGO continues to work in these distinct locales is because of these situated collaborations, partnerships and people. There have been other communities where projects were initiated, but there were not enough vital ingredients for service delivery to get traction. The case studies illustrate that different SLT services will eventuate in different localities, and that positive, reciprocal relationships based on professional respect and valuing of differing stakeholder knowledge and values is imperative (but no guarantee) for successful and ongoing projects.

Innovation for the sake of novelty is unnecessary. We described a Parent Liaison model developed by a parent advocate who identified how she could improve the quality of service by supporting parents in accessing and understanding SLT service delivery. We also described cases of hospital work and community centre work that evolved alongside functioning therapeutic services and teams. SLT was a value-added rather than a new or separate service.

While case 3 works as a model of service delivery, both case 1 and case 2 illustrate that the long-term viability of SLT service provision by an outside provider is questionable. The SLT needs of a hospital would be best met by an integrated SLT department. However, the identified needs of patients must be met to justify the professional presence of speech language therapists on site. We optimistically believe that by demonstrating the need for and benefits of SLT services in hospitals to hospital administrators, other health care professionals will support crucial upward pressure to create a scheme of service for SLTs nationally and create billing structures locally.

In Kenya, despite the initiation of SLT training programmes, increased awareness of the need for SLT services and ongoing SLT service provision by African trained and foreign trained speech language therapists, these 13 years have not seen the development of government funding for SLT positions or services. Professional traction that can allow for growth of SLT service provision to the broader rural Kenyan population is difficult to see. Funding does not drive programme development or service delivery in these collaborations but does place constraints on what is feasible for ongoing growth.

3. How do we ensure that the resources, perspectives and knowledge of all partners is being fully utilised to benefit individuals’ experiencing communication disability?

In these cases, allied health professionals (speech language therapists, occupational therapists) and non-professionals have engaged in models of practice that grew from local understanding about community needs and reciprocal relationships that developed trust over time. The local innovations of Communication Camps and the role of the Parent Liaison Officer demonstrate responsive global engagement by considering the local structural factors and approaching problem solving with global reciprocity. This allowed parents and families to be the experts of their children and their needs and gave them a realistic ability to engage in services in ways that supported their child while accounting for their access to resources and their other responsibilities.

That clinical services exist, have sustained and have developed in Ziwa speaks to the ways that the arrangement is mutually beneficial (e.g. hospital patients receive services, SLT students have placement opportunities). The sustained nature of the partnerships where services are developed alongside each other allows for conversations that interrogate decision-making around practices and necessitate justification of service provision. This is an opportunity to question assumptions and draw on research literature about best practice as part of the cyclical process of acting, reflecting, creating knowledge and changing practice (Hyter, 2014).

The Kilima partnership was able to regroup because there were relationships to rekindle. Both organisations had continued to work in parallel until it was possible to work together again. Solutions come to light in understandings developed during conversations among educators, occupational therapists and speech language therapists, with all team members being willing to respect multiple perspectives to come to collaborative decisions. The team described here is used to considering the resources, time and energy available to explore feasible options for expanding their work. They have moved well beyond any notion of a ‘right way’.

We believe that the development of services in the majority world should be focused on local knowledge, including language and culture. In places like Kenya where SLT services are relatively new and the professionals (even when local or from the region) have been trained by foreigners, the curriculum is often disconnected from local language(s), culture and norms. For example, in the past, the clinical teams described here spent much time talking with parents about play and encouraging dyadic parent-child play, although this is not a typical interaction for Kenyan parents. The East African clinicians believed that if they just kept showing parents the importance of play (as had been taught to them in their training programme), parents would change their behaviour, which led to frustration on both sides.

Duplicating socially and culturally embedded ideas from the minority world about language, interaction and play, and the role of the parent in their child’s early development and education does not result in culturally responsive practice (Hyter, 2014). Speech and language therapists and communication partners of individuals experiencing communication disability need to recognise and acknowledge there are differences across cultural contexts (e.g. Rogoff, 2003) and adapt their recommendations accordingly. As the clinical team described in this article moves along the path from global humility and self-awareness towards global knowledge and reciprocity (Hyter, 2014), they continue to observe and listen to their families, to be able to better adapt and develop more culturally responsive services. Now the team takes a more parent led approach with a focus on daily routines unless the parent indicates a specific interest in engaging their child in play.

Our key message in this work is that to begin to meet the needs of individuals experiencing communication disability in under resourced communities, we need to initiate more creative, nuanced and progressive service models. As aptly stated by Khoza-Shangase and Mophosho (2018), we need to consider ‘next practice’ not just ‘best practice’ (p. 6). As colleagues, we have chosen to take nuanced perspectives of the situation, and each other’s realities, to deeply consider issues of power and control in decision-making. We are not always in agreement about the way to proceed. We work, live and write not from a dichotomous place of right and wrong, good and bad but a space of differing cultures, worldviews and ways of knowing. We strive for global reciprocity, empathy and understanding (and to realise what we do not understand and may never understand) with each other, clients and their families as a way of being together.

Experience and stakeholder feedback have shifted the clinical approaches we have taken over time to attempt more sustainable and locally responsive practices. These case studies provide an opportunity to reflect and to examine the lessons learned from multiple perspectives in real-world contexts (Hyter, 2014) and are not intended to be replicated. That said, we expect that the clinical experiences described here will resonate with speech language therapists in Sub-Saharan African contexts and anticipate that the learnings might be fruitful for thinking about service delivery possibilities in these settings.

For this collaborative team of clinicians, writing together enables us to articulate our current practice and consider what works and what does not. It is a work of knowledge production that has occurred over 13 years. Our lives as an authorship team have changed in large and small ways over this time, as have our clinical skills and our global humility and knowledge (Hyter, 2014) about the lives of families with children experiencing communication disability in Kenya. While our goal is to develop sustainable SLT services for the region, this article is in some ways a small celebration of what is currently being achieved given the limited resources. The collaborations described here have enabled more children and adults experiencing communication disabilities to access services, and those services are now better tailored to meet their needs than they were in the past.

## Conclusion

We need change in the SLT profession to meet the needs of individuals experiencing communication disability globally. Equitable health and education provision, particularly in post-colonial countries where people were historically exploited and/or marginalised to suit those in power, present us with complicated contemporary contexts and situations that have been termed as ‘wicked problems’ (Gwynne & Cairnduff, 2017). The epistemologies, research and curriculums that lay the foundation for SLT practice do not reflect the multilingualism or multiculturalism of the communities many of us work in. Thus, challenges in establishing sustainable, culturally responsive service delivery models are inevitable. Disappointments, failures and struggles are guaranteed, but these too are a part of the story. Knowledge of both the successes and failures is necessary if we seek innovation in service delivery globally.

We offer up these case studies of collaborative practice as contextual realities that may be present in any SLT programming in under-resourced communities. We dispel the idea that success in this work has been linear, progressed on planned timeframes or come to fruition with targeted goal attainment. The fact that our relationships have endured in these communities since 2007 is our primary success. In our work together and our collective histories, there have been notable disappointments, major stumbling blocks and the occasional win. That said, we have impacted individual lives. We have delivered services, organised parent and teacher trainings, provided education about communication disability and alleviated difficulty. However, the positives should not be overstated. If the goal is to provide culturally and linguistically responsive SLT services to every individual experiencing communication disabilities, then we all have a long way to go.
